# Treatment of visceral artery aneurysms and pseudoaneurysms with the use of cerebral flow diverting stents: initial experience

**DOI:** 10.1186/s42155-020-00137-y

**Published:** 2020-09-13

**Authors:** Paolo Rabuffi, Antonio Bruni, Enzo Gabriele Maria Antonuccio, Cesare Ambrogi, Simone Vagnarelli

**Affiliations:** Department of Interventional Radiology, Azienda Ospedaliera San Giovanni Addolorata, Via dell’Amba Aradam 9, 00184 Roma, Italy

**Keywords:** Flow diversion, Flow diverter, Flow diverting stent, Visceral artery aneurysm, Peripheral artery aneurysm, Endovascular aneurysm exclusion

## Abstract

**Background:**

Flow-diverter stents (FDS) are designed to maintain laminar flow in the parent artery and sidebranches and to promote thrombosis of the aneurysm. Although these devices were developed for use in intracranial circulation, FDS could be employed to treat aneurysms regardless of their location, when anatomic factors may limit the efficacy of classic endovascular techniques. The objective of this study is to describe the initial experience of a single center in the treatment of visceral artery aneurysms and pseudoaneurysms (VAA-VAP) with cerebral FDS, analyzing safety, efficacy and 1-year outcome. Between 2016 and 2018 six patients (4 women, mean age 57.6) underwent treatment with FDS of 4 VAA and 2 VAP located in renal (4), hepatic (1) and splenic arteries (1). Mean aneurysm diameter was 14.3 mm (range 8–22). All the aneurysms had sidebranches arising from the neck or had an unfavorable dome-to-neck ratio. Technical success, safety, efficacy and 1-year outcome were analyzed. Follow-ups (FU) with Color-Doppler US and CTA ranged from 12 to 36 (mean 20) months.

**Results:**

Technical success was achieved in all cases. There were no aneurysm rupture nor reperfusion after exclusion. Five out of six (83.3%) FDS were patent at each FU; all the aneurysms showed shrinkage with a mean dimensional reduction rate of 55.8%. Sac thrombosis was observed in 4 aneurysms at 1 (*n* = 3) and at 12-month FUs. There was one sidebranch occlusion with evidence of a small area of kidney hypoperfusion at the 12-month FU, which was asymptomatic. In one patient, a reintervention was needed because CTA showed a severe in-stent stenosis, which was symptomatic. Mean hospitalization was 4.1 days.

**Conclusions:**

Treatment of morphologically complex VAA and VAP with cerebral FDS proved to be safe and efficient. Stronger evidence from larger populations are required.

## Introduction

Visceral Artery Aneurysms (VAA) and Pseudoaneurysms (VAP) are rare clinical entities, which are diagnosed more and more frequently thanks to the increased use of cross-sectional imaging. Despite VAA and VAP are associated with a high incidence of rupture (Loffroy et al. 2015), varying from 3 to 10% depending on their dimensions and location, and that mortality rates following the rupture are reported to be of 20–100%, controversy still exists regarding their treatment (Shanley et al. 1996). In fact, a variety of treatment options are currently available, including open and laparoscopic surgery, and endovascular techniques. Thanks to the reduced invasiveness and to the lower morbidity in comparison to surgery, in the last decade either transcatheter embolization or endovascular exclusion have become the first option for treating VAA and VAP in many centers, especially in patients whose conditions are unfit for open repair (Tulsyan et al. 2007; Kok et al. 2016). However, standard endovascular treatment has limitations: in fact, aneurysm exclusion by coiling may not be appropriate in the case of large-necked aneurysms (Brinjikji et al. 2009); also, the coverage of sidebranching vessels using stent-grafts could lead to an increased risk of end-organ ischemia (Elaassar et al. 2011).

Within intracranial circulation, preserving branch vessel perfusion while excluding aneurysms is mandatory. The application of flow-diversion techniques in the treatment of intracranial aneurysms has represented a revolution in neurovascular interventions: in fact, flow diverter stents (FDS) are specifically designed to maintain laminar flow in the parent artery and sidebranches, while reducing flow velocity within the aneurysm, thus promoting thrombosis of the sac. Although these devices were primarily designed for use in intracranial circulation, cerebral FDS could hypothetically be employed to treat morphologically complex aneurysms, regardless of their location (Sfyroeras et al. 2012). The use of a variety of flow diversion techniques for the treatment of VAA and VAP has recently been reported, with good results in terms of stent patency and aneurysm sac reduction rates (Hardman et al. 2015; Colombi et al. 2018; Abraham et al. 2012; Adrahtas et al. 2016).

The objective of this retrospective study is to describe an initial single-center experience in the treatment of VAA and VAP with cerebral FDS, analyzing safety, efficacy and 1-year outcome.

## Materials and methods

### Patients and aneurysms

The database of patients with VAA and VAP treated in our department was retrospectively researched, and six patients who underwent treatment of VAA (*n* = 4) and VAP (*n* = 2) with cerebral FDS between December 2016 and June 2018 were found. Three of the four VAA were incidentally diagnosed with CTA, and were located in the renal arteries (*n* = 2), the common hepatic artery (*n* = 1) and in a splenic artery branch (*n* = 1). One patient (Pt 2) was diagnosed with VAA after undergoing CTA for right flank pain; the remaining 3 aneurysms were asymptomatic. Two patients (Pts. 5,6) presented with acute flank pain and uncontrollable hypertension: CTA revealed the presence of spontaneous dissections of segmental renal artery branches and circumscribed renal infarctions. Treatment was indicated in these cases because in the following days it was observed that the dissections had undergone morphological changes from stenotic to pseudoaneurysmal configurations.

All of the VAA and VAP either had side branches arising from the neck or had an unfavorable dome-to-neck ratio; mean aneurysm diameter was 14.3 mm (range 8–22) (see Table 1). Informed consent for the procedure and data collection was obtained from all the patients, to whom it was made clear that the use of these stents is currently off-label in the peripheral system.

**Table 1:** Patient demographics

Patient	Age / Sex	VAA /VAPs	Aneurysm size (mm)	Aneurysm location	Symptoms	Follow-up (months)
1	52 / F	VAA	16	Renal	Flank pain	12
2	79 / F	VAA	13	Renal	–	12
3	54 / F	VAA	19	Splenic	–	12
4	65 / F	VAA	22	Hepatic	–	24
5	49 / M	VAP	8	Renal	Flank pain, Hypertension	36
6	47 / M	VAP	8	Renal	Flank pain, Hypertension	24
**Average**	57.6y		**14.3 mm**	**7.3 mm**		**20**

### Indications

In this study, endovascular treatment of VAA was indicated in cases with symptomatic lesions or asymptomatic lesions larger than 1.5 cm. Pseudoaneurysms were treated regardless of their size, since even small VAP (2–5 mm) are at high risk of rupture (Loffroy et al. 2015).

### Procedure

**Fig. 1:**
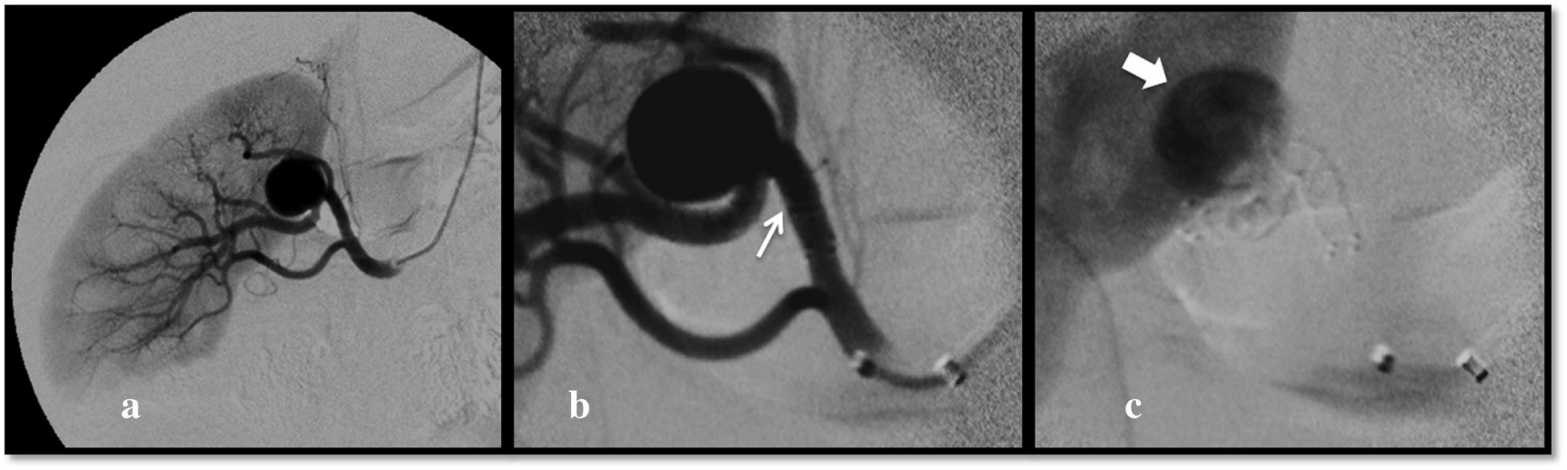
**a**, **b**, **c** (Patient 1) Transhumeral digital subtraction angiography of the right renal artery confirms the presence of a segmental branch VAA (**a**). Postoperative subtracted view immediately after the deployment of a Fred 4/32/26mm (arrow) showing patency of the stent (**b**) and early stagnation (large arrow) of contrast media into the aneurysm sac (**c**)

All procedures were performed by three operators (with respectively 15, 15 and 10 years of experience in peripheral and neurovascular interventions), under conscious sedation, after preoperative preparation with dual antiplatelet therapy (100 mg Aspirin and 75 mg clopidogrel) for 1 week. The renal aneurysms were treated via a transbrachial approach in three out of for cases, and via a transfemoral approach in the remaining case, because of the angulation of the renal arteries, while the treatment of the splenic and hepatic aneurysms was performed via a transfemoral approach. In all cases a 6 Fr guiding-catheter was used to catheterize the renal artery or the celiac trunk, a microcatheter was advanced into the target vessel on a 0.014″ guidewire (Transend, Boston Scientific, Natick, USA), and finally a FDS was deployed (Fig. 1). All patients were discharged under dual antiplatelet therapy for 6 months, to be followed by lifelong prosecution of Aspirin therapy.

The FDS used in this series were the Flow Reduction Endoluminal Device (FRED, Microvention, CA, USA) and the Surpass Streamline (Stryker Neurovascular, Fremont, CA, USA).

### Endpoint

The analyzed endpoints were technical success, safety, efficacy, and the 1-year outcome of the procedure. Technical success was defined as successful deployment of the FDS within the target artery, with documented stent and sidebranch patency at the end of the procedure. Safety was defined as freedom from minor (puncture site haematoma or pseudoaneurysm) or major complications (death, intraprocedural aneurysm sac rupture, acute stent occlusion or foreshortening). Efficacy was defined as stent and sidebranches patency and freedom from aneurysm rupture or reperfusion at 1, 6, and 12 months after intervention. The primary endpoint for outcome consisted of aneurysmal volumetric reduction and the secondary endpoint was sac occlusion at 1-year FU.

### Follow up

Imaging follow-up was scheduled with pre-discharge and 1-month CD-US, followed by CTA scans at 6 and 12 months, and then on a yearly basis. Follow-up of pseudoaneurysms also included a CTA scan at 2 weeks. Follow-up ranged from 12 to 36 months (mean 20 months).

## Results

### Study outcomes

Technical success was achieved in all patients.

**Fig. 2:**
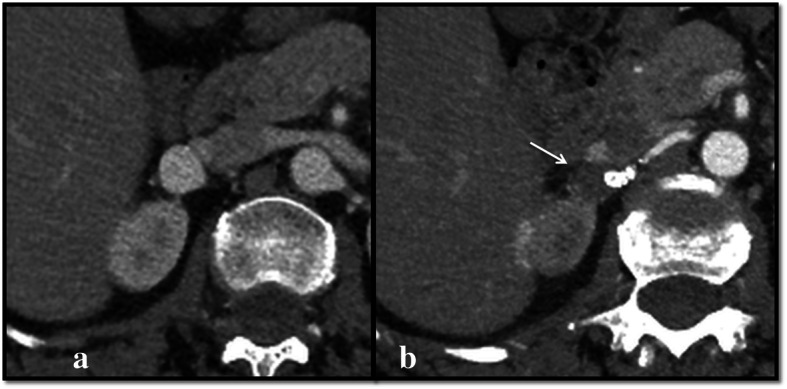
**a**, **b** Axial pre-operative (**a**) CTA scans show the presence of a VAA of the right renal artery (Patient 1), which appears completely excluded (arrow) at the 12-month follow up (**b**)

**Fig. 3:**
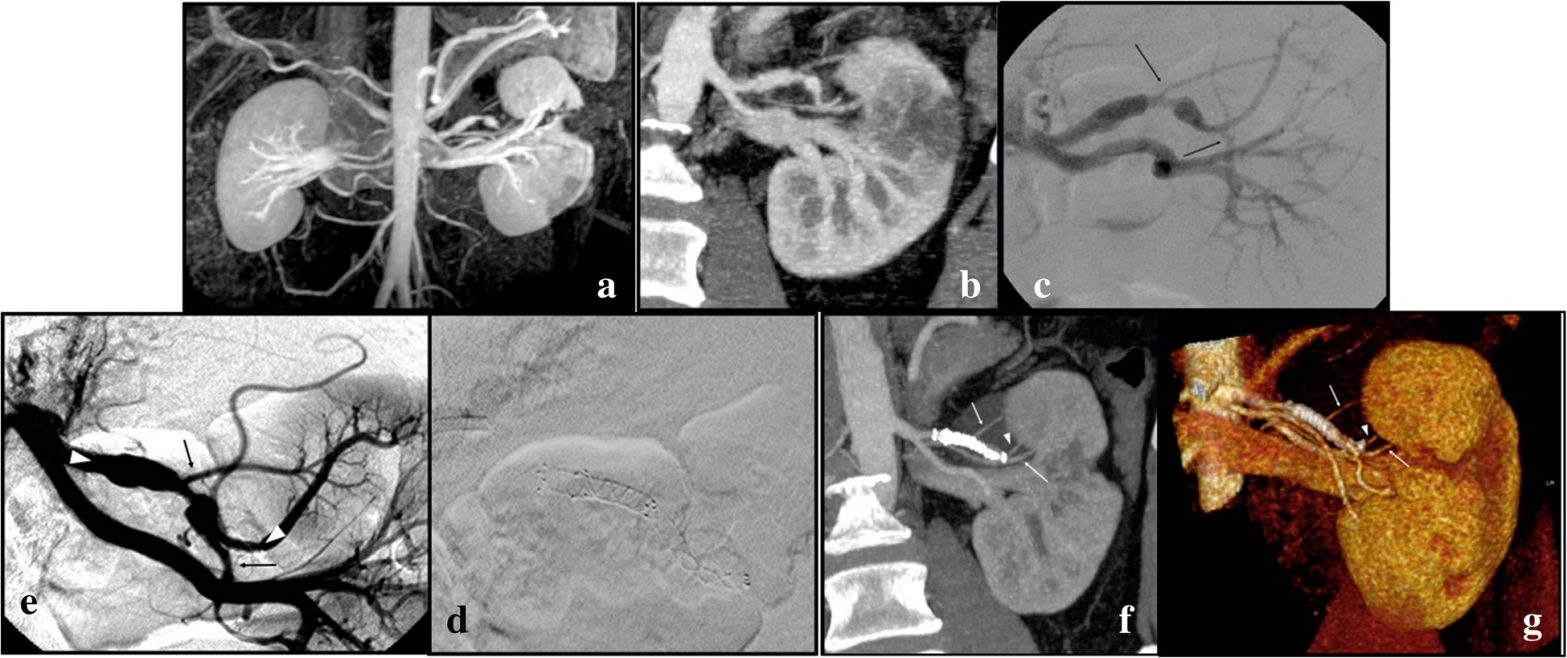
**a**-**g** Coronal CTA (**a**) and 3D MRA imaging (**b**) at baseline, showing a bilobate VAP of the upper branch of the left renal artery and an ipsilateral hypodense-hypointense wedge-shaped parenchymal area of renal infarction. Selective renal artery DSA (**c**–**e**) before and after the deployment of two Fred 4/18/12 mm and 3.5/31/24 mm flow diverter stents, which are placed across two branching vessels (arrows), arising from the pseudoaneurysmatic artery (arrowheads in (**e**) show distal and proximal landing zone of the stents). Coronal CTA and VR imaging (**f**-**g**) at the 12-months follow-up show complete exclusion of the VAP, the parent vessel distally to the FDS (arrowhead) is patent as well as the stented sidebranches (arrows)

**Fig. 4:**
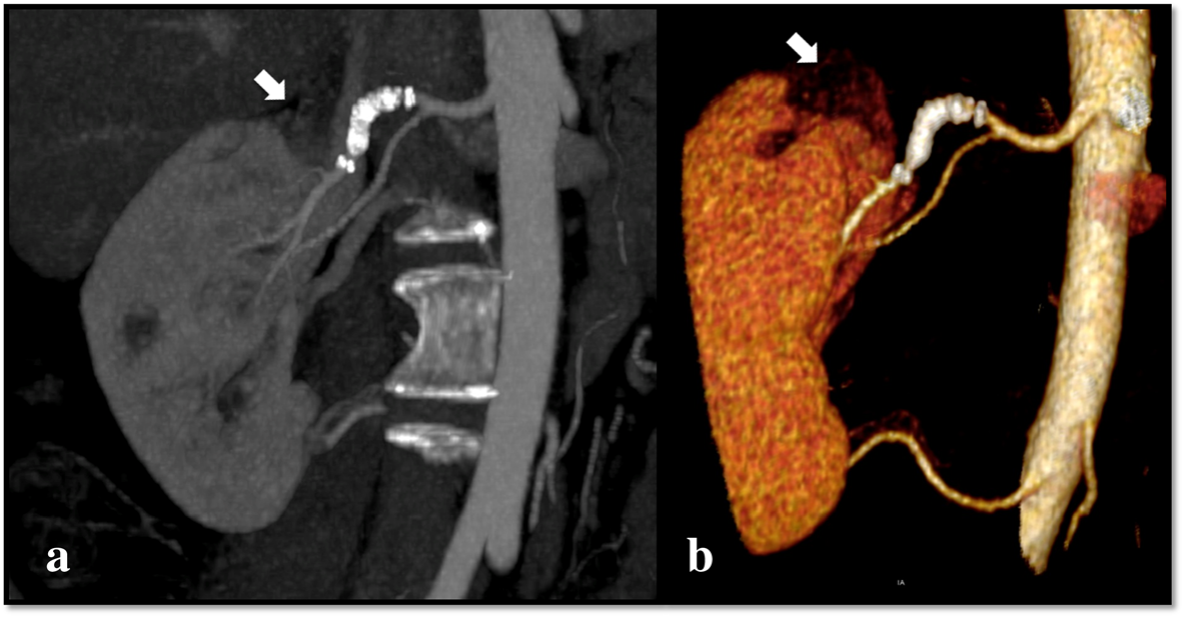
**a**, **b** Coronal CTA (**a**) and 3D volume rendered (VR) imaging (**b**) at 12 months demonstrating developing of complete exclusion of the aneurysm and the patency of the stent. A small area of hypoperfusion in the upper pole of the kidney is visible (large arrow)

**Fig. 5:**
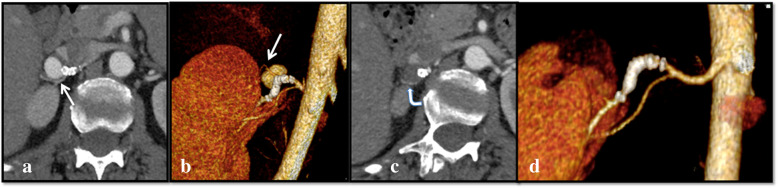
**a**-**d** Coronal CTA (**a**) and 3D VR imaging (**b**) at 6 month FU, showing a patent sidebranch (arrow), which appears smaller in comparison to baseline CT and DSA (see above Figs. 1 and 2), directed to the upper pole of the kidney. **c** and **d** show the same finding at the 12-month FU: the sidebranch ostium is occluded as well as the aneurysm. However, the distal branching vessel is still patent (curved arrow), probably thanks to collateral circulation

- **Efficacy**: Five out of six FDS (83.3%) were patent at each FU; no aneurysm ruptures nor reperfusion after exclusion were observed. Eight out of nine (88.9%) sidebranches covered by the flow diverters were patent at the last FU.
- **Outcome**: Sac shrinkage was observed in all cases with a mean dimensional reduction rate of 55.8% (ranging from 12.5 to 100%). Complete sac thrombosis was observed in two of the four VAA, and occurred respectively at 1 month in one case and at 12 months in another case (see Fig. 2). At the 2 week-FU all of the pseudoaneurysms were excluded from the flow, with no residual contrast opacification of the sac confirmed in the following FU exams.
- **Safety**: In one case (16.7%), because of the recurrence of drug resistant hypertension 40 days after the procedure, a CTA was performed and revealed the presence of a severe in-stent stenosis. In this case because of the bilobate morphology of the VAP, from whose sac originated two sidebranches, it was required to use two partially overlapped FDS. The final angiographic control performed during the first intervention showed a mild in-stent stenosis at the level of the overlapping point, which was considered as not significant, and so a decision not to make an angioplasty was made, also in order to avoid foreshortening of the stents. Therefore, a second intervention was needed, for the correction of the stenosis by angioplasty: after that, the hypertension resolved in a few days. Both the flow diverter stents and the covered sidebranches were patent at the 12 months-FU CTA, which also confirmed the complete exclusion of the pseudoaneurysm (Fig. 3). In Pt 1, a renal branch covered by the FDS underwent occlusion between 6 and 12 months, after the sac was subjected to complete thrombosis, and a small corresponding area of hypoperfusion of the upper pole of the kidney was visible at the last follow-up (Figs. 4 and 5). The patient was completely asymptomatic. No other major or minor complications were observed. Mean hospitalization was 4.1 days (see Table 2).

**Table 2:** Aneurysm details, flow-diverter stents sizes, results and complications

Patient	Location	Stent	Aneurysm size (mm)	1-Year Follow-up diameters (mm)	Sac Exclusion / Age	Shrinkage percentage	Complication
1	Renal	Fred 4/32/26 mm	16	14	Yes / 12 months	12.5%	Sidebranch occlusion
2	Renal	Fred 5.5/32/26 mm	13	11	No	15.4%	–
3	Splenic	Fred 5/26/19 mm	19	9	No	52.6%	–
4	Hepatic	Streamline 5/40 mm	22	10	Yes / 1 month	54.5%	–
5	Renal	Streamline 5/25 mm	8	0	Yes / 2 weeks	100%	–
6	Renal	Fred 4/18/12 mm Fred 3.5/31/24 mm	8	0	Yes / 2 weeks	100%	In-stent stenosis
**Average**			**14.3 mm**	**7.3 mm**		**55.8%**	

## Discussion

Treatment threshold for VAA depends on both the size and the location of the aneurysm: for splenic aneurysms the treatment is commonly indicated in case of lesions larger than 2 cm, while for renal artery aneurysm the threshold is slightly lower (1.5 cm) (Belli et al. 2012; Carroccio et al. 2007; English et al. 2004). However, according to some reports, there is no correlation between renal artery aneurysm diameter and rupture; therefore, since data from published series are scarce and heterogenous, and there is not a recommended standard for indication, a decision to treat should be made on a case by case basis (Yasumoto et al. 2013; Pitton et al. 2015). There are few data comparing surgery with endovascular treatment of VAA, mostly because of the uncommonness of the condition; however a reduction in complication rates, hospitalization time and overall cost has been observed with endovascular techniques (Hislop et al. 2009), which also have been shown to have excellent early and midterm outcomes (Etezadi et al. 2011). Among endovascular options, coil embolization and stent graft exclusion are the most frequently utilized techniques. Nevertheless, embolization with coils requires sac catheterization and is not feasible in the case of unfavorable large aneurysm neck or in the presence of sidebranches arising from the neck or the aneurysmal sac itself. Moreover, intrasaccular maneuvers are not safe in the case of pseudoaneurysms, and may lead to intraprocedural rupture of the aneurysm which is a life-threatening complication. Furthermore, aneurysmal exclusions with covered stents may affect sidebranches perfusion¸ potentially causing end-organ ischemia; also, stent-grafts tend to have a large and stiff profile making their use in smaller and more tortuous vessels potentially more difficult (Murray et al. 2019).

An ideal device for visceral aneurysms repair should have a low profile and be flexible enough to be deployed in difficult anatomies; furthermore, it should also achieve aneurysm exclusion avoiding the risk of sac catheterization and potential rupture, even in presence of large, unfavorable aneurysm necks, while preserving existing sidebranches patency. The option of flow-modulation as a treatment tool aimed at excluding aneurysms, while preserving the patency of the parent artery and sidebranches, was the strategy behind using Cardiatis Multilayer Stent (Cardiatis, Isnes, Belgium), which is a cobalt self-expandable stent specifically approved for visceral and peripheral artery aneurysm repair. Early reports showed encouraging results with stent patency rate of 89% and aneurysm exclusion rate of 84% at 1-year follow-up (Ruffino et al. 2012). However, mid-term results were judged unsatisfactory, since the data showed a drop in stent patency rates (60% at 2-years) and safety concerns were raised after a case of disconnection at 2 years was reported (Balderi et al. 2013; Ferrero et al. 2013). Cerebral FDS, on the other hand, have unique characteristics which make these device close to the definition of the “ideal tool”: in fact the greater metal coverage of these stents gives them a design that promotes slow and progressive thrombosis of the aneurysm by reducing the flow at the aneurysm neck, disrupting aneurysm influx and efflux and creating a turbulence which leads to an increased blood viscosity within the sac (Seshadhri et al. 2011). It has been shown that the endovascular mesh operates as a frame for endothelization, jailing the aneurysm neck and resulting in angiographic aneurysm exclusion (Kallmes et al. 2007). When an FDS is placed across a sidebranch or a perforator, the laminar flow into these vessels is preserved through the stent interstices, as long as a pressure gradient persists (Bhogal et al. 2017). Although the flow-modulation mechanism leads to earlier sac depressurization, the time required to achieve aneurysm exclusion, in comparison to conventional techniques, is longer, in terms of weeks to months (Sfyroeras et al. 2012; Dholakia et al. 2017; Leonardi et al. 2011). However, rather than the thrombosis, the most predictive effect of clinical success is the dimensional reduction of the sac, which is the result of aneurysm depressurization (Sfyroeras et al. 2012). Nonetheless, in the majority of published series, aneurysm sac thrombosis was evaluated primarily and the aneurysms were shown to undergo progressive exclusion over the following six to 12 months, but the rate and degree of thrombosis were inconstant (Lylyk et al. 2009). Recently published results from the Fred Italian Registry Follow-up have shown complete or nearly complete occlusion of the aneurysm in 94% of cases at 3–6 months, increasing to 96% at 12–24 months’ follow-up. The use of cerebral FDS for the treatment of hepatic, renal and other VAA and VAP has been reported since 2012, with good clinical outcomes (Hardman et al. 2015; Colombi et al. 2018; Abraham et al. 2012; Adrahtas et al. 2016; Maingard et al. 2019; Shlomovitz et al. 2011). Similarly to the results of those reports, in this series the stent primary and assisted primary patency at 1-year were 83.3% and 100%, and all aneurysms showed dimensional reduction varying from 12.5 to 100% (mean 55.8%). Eight out of nine sidebranches (88.9%) arising from the aneurysm and covered by the flow diverters were patent at the 1-year follow up. One segmental renal branch underwent ostial occlusion between 6 and 12 months, as a result of aneurysm sac thrombosis, and the patient developed a small asymptomatic ischemia of the upper lobe of the kidney (see Figs. 4 and 5). However, the extent of the organ ischemia was smaller with respect to the size of the occluded branch: this could be explained by the presence of a collateral circulation, which progressively lowered the pressure gradient necessary for maintaining a direct flow into the sidebranch.

The pseudoaneurysms treated in this series were a consequence of acute spontaneous renal artery dissection, which affected the renal arteries at the level of the segmental bifurcation. The clinical manifestation was the combination of severe flank pain and uncontrollable hypertension. Additionally, in these cases, baseline CTA showed the presence of circumscribed renal infarctions. The decision was made to use an FDS for the treatment in order to achieve flow remodulation without excluding the segmental sidebranches, and potentially worsening renal perfusion. For this reason, it was not considered appropriate to use either stent-grafts or coronary stents, which would have not allowed the exclusion of the pseudoaneurismatic sac from the flow, and would furthermore have had a stiffer profile than an FDS.

When using an FDS for treating a visceral aneurysm, the operator must take into account that this kind of stent requires a necessary learning curve for non-neurointerventionists; in fact the deployment mechanism of the device is not based on the classical pull-back stent movement; rather, a combination of push-forward and pull-back (“push and pull”) techniques may be required, because FDS have low radial opening forces (Dmytriw et al. 2019). It is also necessary that the device be correctly sized during preprocedural planning, because undersizing of the FDS may cause inadequate wall apposition of the device and incomplete coverage of the aneurysm neck, which may compromise aneurysm occlusion (Estrade et al. 2013; Mut and Cebral 2012). On the other hand, oversizing of the device could alter the hemodynamic properties of the FDS, possibly leading to in-stent stenosis (Kellermann et al. 2019). Since these stents are specifically designed for intracranial circulation, the maximum available diameter is 5.5 mm, thus the treatment can only be proposed for vessels with a maximum caliber of 5 mm. Furthermore, since FDS have greater metallic surface coverage, with higher porosity in comparison to traditional bare stents, they are burdened by a higher incidence of thrombosis, up to 8.3% at 30-days (Sfyroeras et al. 2012), therefore double antiaggregation is mandatory. Finally, the high cost of these devices must be taken into account, inasmuch as the use of the cerebral FDS in the peripheral system is off-label, thus healthcare reimbursement may not cover the full cost of the product.

However, there are several clear advantages to using flow diversion techniques: the aneurysmal artery is treated at the neck, which is the point most at risk of future recurrence, sidebranches patency is preserved and, finally, the risk of incidental rupture associated with aneurysm sac catheterization and intrasaccular maneuvers are avoided.

## Conclusion

The exclusion of VAA and VAP may represent a challenge because of the technical difficulties associated with the treatment. Although there are relatively few reports as yet in the literature, endovascular repair of VAA and VAP with cerebral FDS seems to be a reliable option. Despite the greater amount of time required to obtain the shrinkage and the occlusion of the aneurysms, in comparison to traditional endovascular techniques, treatment with FDS in this series proved to be safe and efficient, and provided the possibility of overcoming some of the limits of the currently available techniques. Obviously, more robust evidence from larger population studies and prospective studies are required to reinforce these initial indications.


**Additional file 1**

## Data Availability

All data and materials related to the study are available if requested.
